# Oral Health Care Among Women in Perimenopause or Menopause: An Integrative Review

**DOI:** 10.1111/jmwh.13668

**Published:** 2024-07-24

**Authors:** Namitha Thomas, Kath Peters, Kate O’ Reilly, Mariana S. Sousa, Ajesh George

**Affiliations:** ^1^ Australian Centre for Integration of Oral Health Ingham Institute for Applied Medical Research Liverpool New South Wales Australia; ^2^ School of Nursing and Midwifery Western Sydney University Penrith New South Wales Australia; ^3^ Improving Palliative, Aged and Chronic Care through Clinical Research and Translation, Faculty of Health University of Technology Sydney Ultimo New South Wales Australia; ^4^ School of Dentistry Faculty of Medicine and Health The University of Sydney Camperdown New South Wales Australia; ^5^ School of Nursing Faculty of Science, Medicine & Health University of Wollongong Wollongong New South Wales Australia

**Keywords:** attitudes, health care providers, knowledge, menopause, oral health, practices, perimenopause, women

## Abstract

**Introduction:**

Women in menopause are at a higher risk of developing oral health problems, affecting their overall quality of life. Several studies have identified the role of health care providers in addressing women's oral health needs across various phases of their lives, yet a review in the area of perimenopause and menopause has not been undertaken. Therefore, the aim of this review was to explore current evidence regarding the oral health knowledge, attitudes, and practices of women in perimenopause or menopause and their health care providers. Additionally, guidelines and recommendations to inform strategies for oral health promotion are included.

**Methods:**

A systematic search was carried out across 5 databases. Inclusion criteria included articles published in English that examined at least one study outcome: oral health knowledge, attitudes, and practices of either women in perimenopause or menopause or of health care providers or guidelines around oral health care. Qualitative, quantitative, mixed‐methods, and experimental studies with survey components were included with no restrictions on publication period, quality, or setting.

**Results:**

A total of 12 articles met the inclusion criteria, with a majority being of poor quality and mostly from low‐income and middle‐income countries. Overall findings indicated that there was a lack of knowledge and limited practices in maintaining oral hygiene and visiting the dentist among women in perimenopause or menopause. Health care providers exhibited poor attitudes in advising the importance of periodic dental check‐ups and informing oral health changes during this period. There were also insufficient guidelines to adopt care for women and guide health care providers in their practice.

**Discussion:**

Women in perimenopause or menopause have limited oral health knowledge and unmet oral health needs. Appropriate guidelines and supportive strategies are required to assist health care providers in providing comprehensive care and encouragement to women in perimenopause or menopause to improve their oral health.

## INTRODUCTION

Menopause is a significant event in a woman's life, marking the end of her reproductive years.^1^ By 2030, it is estimated that the global population of women in menopause will reach 1.2 billion.^2^ During this transitional period, women often experience various adverse symptoms, including hot flushes, night sweats, urinary disturbances, mood changes, depression, and declines in cognition, as well as bone and joint pain.^3^ Additionally, menopause has been associated with unpleasant oral health changes such as burning sensations of the mouth, dryness of mouth, alterations in taste, inflammation of supporting tissues of the teeth, osteoporosis of the jaws, and an increase in tooth decay.^4^, ^,5^ These oral health conditions can significantly affect quality of life.^6^ However, there is currently limited evidence on the knowledge, attitudes, and practices of women in perimenopause or menopause regarding oral health. The few studies focusing on this area have mainly explored the systemic aspects of perimenopause or menopause and oral health.^7^, ^8^ The only comprehensive review conducted to date has focused on oral health changes and its management in menopause.^9^ Providing appropriate assistance during the early period of the menopausal transition may help to reduce the prevalence of oral health problems, improve quality of life, and support a healthy menopausal life. Studies have identified that primary and women's health care providers, such as midwives, could play a key role in promoting oral health among women in perimenopause and menopause.^10^ However, this aspect of women's health care has also not been extensively reviewed in the literature.

**QUICK POINTS**

✦ There is very limited research addressing the oral health needs of women in perimenopause or menopause and the practice of their health care providers.
✦ Women in perimenopause or menopause have limited awareness and practices regarding oral health care.
✦ Health care providers have not provided sufficient guidance to women in managing oral health during this period.
✦ There are insufficient practice guidelines to support health care providers in promoting oral health among women in perimenopause or menopause.
✦ High‐quality studies and supportive strategies are needed to improve the oral health of women and guide health care providers in their practice.

### Background

Menopause is a physiologic process characterized by the cessation of the menstrual cycle and is caused by the progressive depletion of ovarian follicles.^11^ It usually occurs in women between 45 and 55 years of age. Menopause can also be surgically induced by ovarian surgery (oophorectomy) or treatment induced with radiation therapy, chemotherapy, and other medications.^12^ Menopause involves 3 stages: perimenopause, menopause, and postmenopause.^13^ Perimenopause describes the menopausal transition phase characterized by continuous changes in the menstrual cycle over the past 11 months, whereas menopause refers to those women who have had a final menstrual period. Postmenopause is the period when a woman has not had a menstrual cycle for 12 months or more.^13^ The symptoms of menopause can negatively impact various aspects of women's lives including systemic, physical, and psychological domains.^3^ Interviews of Emirati women found that vasomotor symptoms and weight gain during menopause led to anxiety, depression, insomnia, and memory loss.^14^ For most, symptoms begin mildly and increase in intensity in the later years of the menopausal transition,^3^ impacting on women's quality of life.^15^ As their life expectancy has increased, women may experience poorer physical and mental health for an extended period of time.^16^

The estrogen variations leading to menopause can also lead to oral health problems.^4^, ^5^ A cross‐sectional study found that women who have periodontal problems during postmenopause have an impaired quality of life compared with women in postmenopause with healthy a periodontium.^17^ The mouth is an organ that supports the process of eating, chewing, and swallowing but also contributes to individuals’ appearance and socialization.^18^ Therefore, any imbalance in oral health functioning can affect the daily activities of individuals leading to poor quality of life.^19^

Studies have highlighted numerous barriers women face in managing oral health problems in various phases of their life. These primarily include affordability and accessibility of dental services, previous negative experiences, time constraints, lack of priority, lack of knowledge, lack of policies, and cultural barriers.^20^, ^21^ There is limited evidence, however, describing women's experiences in accessing dental care during menopause. In a 2020 cross‐sectional survey of 1115 women in menopause and postmenopause, Singh and Jamwal identified safety concerns, lack of awareness, time constrictions, and lack of priority as barriers in managing oral health.^22^

Studies have shown that health care providers such as primary care providers, gynecologists, nurses, and midwives play an essential role in addressing the unique health care needs of women.^10^ This includes promoting oral health among women across their life span. Evidence has shown that health care providers can be effective in improving the oral health outcomes of pregnant women.^23^, ^24^ Additionally, these models of care are suitable and cost‐effective to implement into practice.^25^, ^26^, ^27^ Numerous challenges have been cited, however, such as time constraints, high workload, and limited interest and knowledge in oral health practices.^28^, ^29^ Most literature has focused on pregnant women,^30^, ^31^ with very few studies investigating the importance of managing the oral health of women in menopause.^32^

It is evident that women in menopause are at high risk of developing oral health problems, yet no comprehensive review has been undertaken to further explore this area. Currently, it is unclear whether additional barriers exist for women in menopause to maintain oral health and for health care providers to play a role in promoting oral health in this population. Gathering this information will aid in identifying gaps in service delivery and provide a road map to develop tailored preventive strategies to promote good oral health for women approaching menopause, thereby contributing to their overall health and well‐being.

### Aim

The aim of this integrative review was to synthesize current evidence regarding the oral health knowledge, attitudes, and practices of women in perimenopause or menopause and their health care providers. It also aimed to explore the current guidelines and recommendations for oral health promotion among women during this period.

The following research questions further guided the review: (1) What are the knowledge, attitudes, and practices of women in perimenopause or menopause toward oral health care? (2) What are the knowledge, practices, and perceptions of health care providers toward oral health care for women in perimenopause or menopause? (3) What are the current guidelines and recommendations to promote oral health for women in perimenopause or menopause?

### Definition of Terms

There are inconsistencies in the literature regarding the various terminologies used to describe the menopausal period^33^ when compared with the Stages of Reproductive Aging Workshop staging system,^13^ which is widely considered as the gold standard for defining the various stages of menopause. In this study, the term *women in perimenopause or menopause* has been used to include any women who exhibit hormonal fluctuation, anovulatory cycles, and the onset of cycle irregularity and symptoms, or those who have experienced a complete cessation of their menstrual cycles. Likewise, the term *menopause* includes the postmenopausal period.

*Woman* or *women* is defined based on biological sex rather than gender identity. This includes individuals who were born with ovaries, fallopian tubes, and a uterus as anatomical reproductive structures.

The term *health care providers* refer to various health care staff that women come into contact with during perimenopause or menopause other than oral health professionals and include (but not limited to) obstetric and gynecology or primary care physicians, midwives, nurse practitioners, and nurses.

#### Knowledge, Attitudes, and Practices

Knowledge includes awareness of the association between perimenopause or menopause and oral health, complications, and the impact of prescribed medication on oral health, health risks of poor oral health, and knowledge on seeking out oral health resources and services for the management of oral health problems during this period.

Attitudes refer to a person's perception toward oral health, perceived barriers to accessing oral health services, and perceptions toward health care providers engaging in oral health promotion activities. It also refers to the attitudes of health care providers toward promoting oral health among women in perimenopause or menopause, including the acceptability and feasibility of this role.

Practices include the actions that a person engages in to maintain oral health such as tooth brushing frequency, type of aid used, and dental visits. It also refers to oral health promotion activities engaged by health care providers.

## METHODS

**Figure 1:**
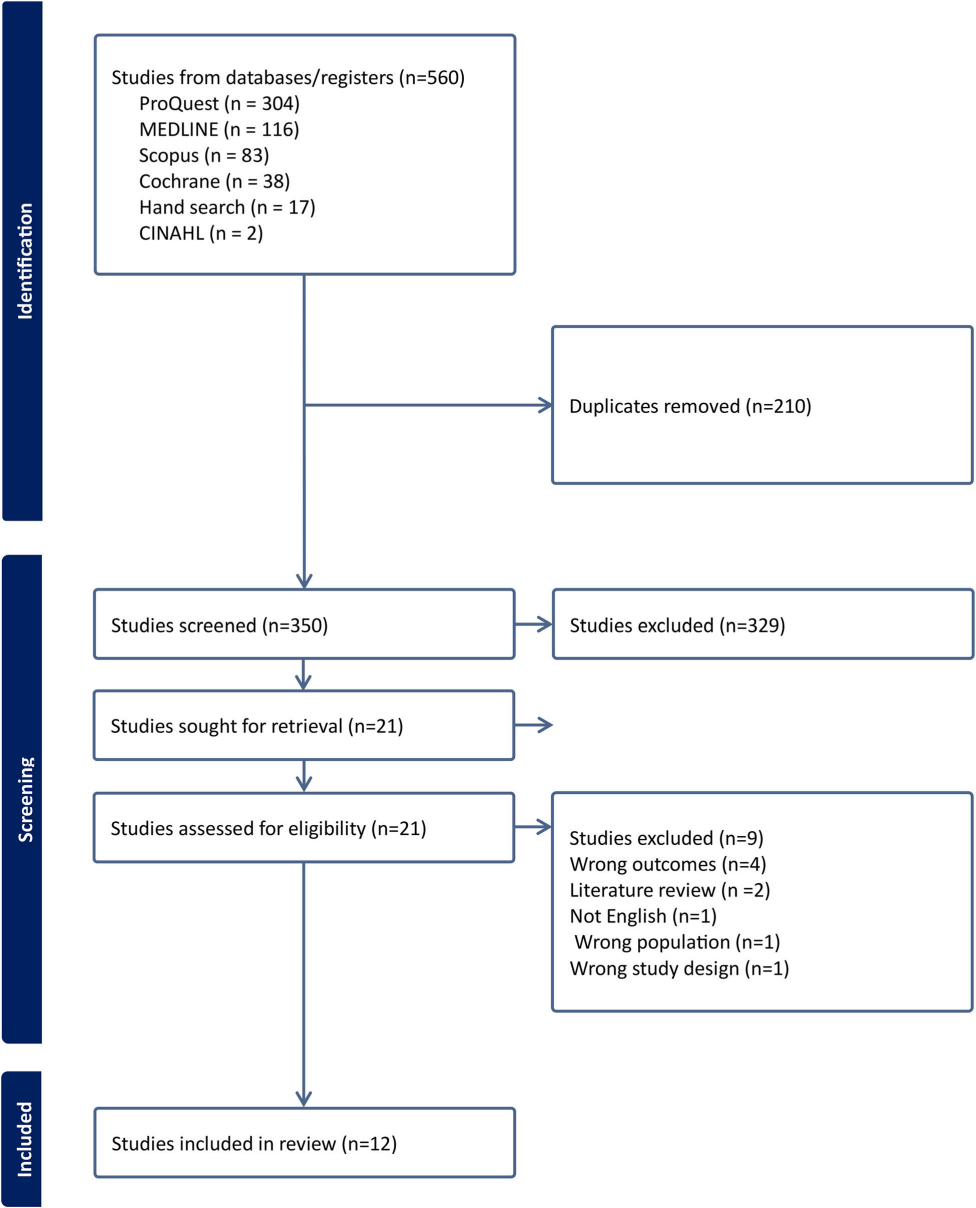
Preferred Reporting Items for Systematic Reviews and Meta‐Analyses Flow Chart of the Study Selection Process

Due to the limited research in this area, it was important to review both quantitative and qualitative studies to explore current evidence addressing the research questions. Thus, an integrative review methodology was chosen because it enables integration of diverse study designs, assesses the quality of the evidence, and identifies knowledge gaps.^34^ This review followed the integrative review methodology suggested by Whittemore and Knafl,^35^ which is a staged process that includes problem identification, literature search, data evaluation and analysis, and finally reporting of findings. The Preferred Reporting Items for Systematic Reviews and Meta‐analysis framework^36^ were used to report the findings in this integrative review (see Figure 1). The review protocol was registered in PROSPERO (CRD42023416503). Institutional review board approval was not necessary for this review as it did not involve direct data collection from human participants.^37^

### Eligibility Criteria

Inclusion criteria included articles published in English that assessed at least one study outcome (knowledge, attitudes, and practices) of women in perimenopause or menopause or their health care providers’ knowledge, attitudes, or practices toward oral health, guidelines, or recommendations regarding oral health for women in perimenopause or menopause. All qualitative, quantitative, and mixed‐methods studies were included along with any experimental studies that had a presurvey component. No restrictions were placed on the time of publication, quality, or location of the study.

### Data Sources, Search Strategy, and Study Selection

A preliminary search was undertaken using one database (MEDLINE) to identify keywords and develop search strategies in consultation with a university librarian from the relevant fields of interest. Following this, a search was undertaken across 5 databases (MEDLINE, CINAHL, Cochrane, ProQuest, and Scopus) using various search strategies. Some of the keywords used included *menopause*, *perimenopause*, *post menopause*, *menopausal complaints*, *oral health*, *dental care*, *oral hygiene*, health care*, *health care providers*, *doctors*, nurs*, *interprofessional*, *knowledge*, *perceptions*, *attitudes*, *awareness practices*, *barriers*, *facilitators*, *guidelines*, *recommendations*, *management*, and *suggestions*. Subject headings, Boolean modifiers, and Boolean operators (AND, OR) were used to assist with the search strategy and to combine the search terms. The reference list of the key articles was also hand searched. Articles that matched the inclusion criteria were organized using the EndNote referencing software and then imported into Covidence for screening. Duplicates were removed, abstract‐title screening was performed by 3 investigators (N.T., K.O.R., A.G.), and reviewing of the selected full text was performed by 2 investigators independently (N.T., A.G.). Any discrepancies related to screening were resolved by discussions with a third investigator (K.O.R.). A total of 12 studies met the inclusion criteria and were included in this review.

### Data Extraction and Data Synthesis

Relevant information extracted from the selected studies included the author, year of publication, the country where the study was conducted, study setting, sample size, and age group (Table 1).

**Table 1:** Summary of Included Studies

Author Year of Publication Country	Design and Method	Design and Sample (Age)	Results: Knowledge, Attitudes, and Practices or Guidelines	Quality Scoring^a^
**Women**				
Yakar et al^32^ 2021 Turkey	Cross‐sectional surveys, Department of Periodontology, Faculty of Dentistry, Ege University, Izmir	115 women in premenopause and postmenopause (35‐65 y)	*Practices* Oral hygiene habits and aids—Brushing frequency (postmenopausal women): 59%, brushed ≥2 times/d; 34%, brushed once; 7%, brushed less than once Dental visits (postmenopausal women): 71%, visited over a concern; 14%, visited in a year; 9%, visited in 2 y	High
Singh and Jamwal^22^ 2020 India	Cross‐sectional surveys, Outpatient Department of Periodontics and Oral Implantology, Institute of Dental Science, Jammu and Kashmir	1115 women in menopause and postmenopause (46‐80 y)	*Knowledge* Risk factor: 22%, menopause can cause the receding of gums; 18%, menopause can cause loss of teeth; 8%, poor oral hygiene can cause the loosening of gums Preventive oral hygiene measures influencing oral health: 19%, tooth cleaning prevents the loosening of teeth	Low
			*Attitudes* Consulting dentist: 80%, willing to revisit the dentist; 16%, less interest; 4%, not willing Importance of oral health: 91%, believed oral health can be ignored Barriers to accessing dental services: 46%, time; 33%, priority; 15%, advice not to seek dental treatment; 7%, safety concerns	
			*Practices* Oral hygiene habits and aids—Brushing frequency: 6%, brushed more than twice; 30%, brushed less than twice; 62%, brushed once; 1%, brushed irregularly Oral hygiene habits and aids—Frequency of changing toothbrush: 3%, less than a month; 2%, monthly; 14%, every trimester; 18%, every 3‐6 mo; 27%, quarterly; 23%, between 6‐8 mo; 16%, greater than a year Oral hygiene habits and aids—Various types: 76%, used toothbrush and toothpaste; 18%, used toothpowder; 4%, used Datun; 1%, used Manjan Oral hygiene habits and aids—Frequency of using a mouth rinse: 5%, used more than twice; 17%, used twice; 43%, used once; 34%, never used Oral hygiene habits and aids—Frequency of rinsing after meals: 61%, rinsed mouth after meals; 35%, occasionally; 4%, do not rinse Dental visits: 5%, last week; 9%, last month; 17%, between 3‐6 mo; 21%, between 6‐12 mo; 46%, less than a year; 2%, never	
Hameed and Radhi^38^ 2023 Iraq	Cross‐sectional surveys, Medical City Teaching Hospital, Baghdad City	90 women in menopause (45‐65 y)	*Knowledge* Preventive oral hygiene measures (study group): 100%, aware clean mouth prevents tooth decay	Moderate
			*Attitudes* Consulting dentist: 100%, agreed periodic visits are important Importance of oral health: 100%, agreed women are responsible for maintaining a healthy mouth	
			*Practices* Dental visits: 100%, had visited the dentist Dental visits—Reasons: 24%, pain; 24%, extraction; 31%, filling; 4%, decay; 16%, others	
Basu et al^39^ 2021 India	Cross‐sectional surveys, secondary care hospital, North‐western district of Delhi	136 women with perimenopause symptoms or with menopause (40‐59 y)	*Practices* Oral hygiene habits and aids—Brushing frequency: 16%, brushed twice daily; 84%, once daily Oral hygiene habits and aids—Types: 98%, toothbrush and toothpaste; 22%, wooden toothpick; 10%, chewstick; 4%, dental floss Dental visits: 21%, <6 mo, 20%, between 6‐12 mo, 16%, between 1‐2 y, 9%, between 2‐5 y, 16%, >5 y, 18%, never received oral health service Dental visits—Reasons: 38%, consultation; 53%, pain; 10%, revisits for existing ailments	Moderate
Qasim et al^40^ 2017 Iraq	Cross‐sectional surveys, College of Dentistry, University of Mosul and private clinics	120 women in premenopause (30‐45 y) and postmenopause (46‐61 y)	*Knowledge* Preventive oral hygiene measure—postmenopausal women: 32%, poor oral hygiene habits contribute to periodontal problems	Low
			*Practices* Oral hygiene habits and aids—Brushing frequency: 20%, brushed more than once; 26%, brushed twice daily; 54%, brushed once daily Oral hygiene habits and aids—Types: 3%, used 2 or more aid; 9%, used stick; 6%, used Miswak; 82%, used toothbrush Dental visits: 14%, visited the dentist frequently; 86%, irregular visits	
Deepa and Jain^41^ 2016 India	Cross‐sectional surveys, Periodontology Outpatient Department, Subharti Dental College and Hospital, Meerut	90 women in menopause (45‐80 y)	*Practices* Oral hygiene habits and aids—Brushing frequency: 6%, never brushed	Low
Gatchalian et al^42^ 2022 Philippines	Cross‐sectional surveys, educational institutions in Cavite	42 women in menopause (40‐60 y)	*Knowledge* Risk factors: 53%, menopause as a risk factor for causing oral health problems	Low
			*Attitudes* Consulting dentist: 69%, good oral health‐seeking behavior	
Palomo et al^43^ 2013 United States	Cross‐sectional surveys, Centre for Specialized Women's Health at the Cleveland Clinic Foundation	94 women in postmenopause	*Knowledge* Risk factors: 1%, agreed risk of tooth loss in menopause; 4%, increased risk of gum‐related problems in menopause	Low
			*Practices* Dental visits: 86.2%, visited every 6 mo; 3.2%, visited every 2 mo; 10.6%, did not know	
**Health care providers**				
Rashidi Maybodi et al^44^ 2018 Iran	Cross‐sectional surveys	40 obstetricians (32‐73 y)	*Knowledge* Symptoms of poor oral health: 70%, xerostomia as a symptom; 33%, increased incidence of gingivitis; 18%, taste alteration; 38%, burning mouth; 3%, decreased salivary flow; 70%, thinning of the mucosa; 10%, not aware of oral health changes Oral health risk factors: 35%, menopause responsible for the mobility of teeth	Low
Patil et al^45^ 2012 India	Cross‐sectional surveys, health care providers practicing in public and private medical colleges in Bangalkot district	73 gynecologists	*Knowledge* Oral health risk factors: 98%, hormonal changes cause changes in the gingival tissue	Low
			*Attitudes* Treatment of oral symptoms: 47%, gingival changes should be treated; 53%, subsides automatically Periodic dental check‐ups: 18%, would insist; 52%, would not insist; 31%, will advise not insist	
			*Practices* Treatment strategies: 61%, prescribed mouthwashes, gels, antibiotics, and analgesics; 39%, prescribed vitamin supplements	
Matsuki et al^46^ 2013 Japan	Cross‐sectional surveys, 380 medical institutions		*Knowledge* Oral health risk factors: 82%, aware hormonal changes cause oral symptoms; 9%, not aware; 9%, did not respond	Low
			*Attitudes* The need for interprofessional collaboration—Physician response regarding cooperation: 63%, physician agreed cooperation with dentist necessary; 33%, no idea about this; 4%, felt unnecessary Nurses response: 57%, cooperation with dentist is necessary; 43%, no idea about this Physician response regarding dentist referral: 4%, dentists referred frequently to medical professionals; 20%, occasionally; 76%, never	
			*Practices* Frequency of reporting oral symptoms: 14%, patients informed about oral symptoms frequently; 65%, occasionally; 79%, a combination of both; 19%, never; 2%, did not answer Prevalence of poor oral health: 80%, dry mouth; 60%, taste disorder; 40%, burning sensation in the mouth; 20%, temperomandibular joint pain Treatment strategies: 59%, referred to a specialist; 48%, mentioned in the health care record; 44%, informed patients about oral health changes in menopause; 15%, prescribed medication; 7%, checked for Sjögren syndrome; 3%, did not provide any guidance; 0.7%, stopped medication; 0.7%, advised lifestyle guidance	
**Guidelines**				
Shifren and Gass^47^ 2014 United States	Guidelines for health care providers for the care of women in midlife		*Guidelines for health care providers for the care of women in midlife* Women in menopause should maintain good oral hygiene Use toothpaste and mouth rinses containing fluorides Women should undergo regular dental and periodontal examinations Inform dental care providers of the findings of bone mineral density testing to maintain dental and periodontal health Inform dental care providers about the use of relevant medications	Moderate

^a^ Scoring: High (80%‐100%), moderately (50%‐79%), low (<50%).

A thematic synthesis approach^48^ was used for synthesizing the study findings. This 3‐staged approach initially involved reading the findings and coding them line‐by‐line according to the meaning and content. This was undertaken by one reviewer (N.T.) using both inductive and deductive approaches to develop the initial codes. These codes were then grouped based on similarities and dissimilarities into themes that were then reviewed by a second reviewer (A.G.) and revised accordingly. The final step involved a consensus meeting with the team to explore interpretations and finalize the themes. The themes were generated aligning to the research questions. Quantitative data presented in narrative format along with any direct quotes were used to support the themes (Table 1).

### Quality Assessment

The Joanna‐Briggs Institute (JBI) critical appraisal checklist^49^ and Agree II checklist^50^ were used to assess the methodological quality of the articles and guidelines, respectively. The JBI checklist varied according to the type of studies assessed.^49^ The quality of these studies was assessed using a scoring system (one point for each applicable item) and was carried out by 2 authors (N.T. and K.P.). Two authors (M.S., A.G.) were consulted to resolve any discrepancies in the quality assessment scoring. Once consensus was achieved, the overall quality was rated as high (80%‐100%), moderate (50%‐79%), and low (<50%) using cutoff values^51^, ^52^ (Table 1).

## RESULTS

Twelve studies were identified that addressed the study research questions.^22^, ^32^, ^38^, ^39^, ^40^, ^41^, ^42^, ^43^, ^44^, ^45^, ^46^, ^47^ The studies were published between 2011 and 2023 with a majority conducted in India (n = 4), Iraq (n = 2), and the United States (n = 2). For the first research question, 8 articles were identified involving women in perimenopause, menopause, and postmenopause (n = 1610).^22^, ^32^, ^38^, ^39^, ^40^, ^41^, ^42^, ^43^ Three articles were reported for the second research question involving health care providers (n = 113).^44^, ^45^ One study did not provide the number of health care professionals and consisted of 380 medical institutions.^46^ All studies involved cross‐sectional surveys. Most studies focused on oral health practices (n = 9)^22^, ^32^, ^38^, ^39^, ^40^, ^41^, ^43^, ^45^, ^46^ and knowledge (n = 8),^22^, ^38^, ^40^, ^42^, ^43^, ^44^, ^45^, ^46^ and very few focused on attitudes (n = 5).^22^, ^38^, ^42^, ^45^, ^46^ There were some overlaps, with some studies (n = 7) ^22^, ^38^, ^40^, ^42^, ^43^, ^44^, ^45^, ^46^ focusing on more than one area. Only one guideline was identified for the third research question, which was from the United States.^47^ The majority of studies were considered low quality (n = 8), and only one was high quality. The main themes identified were knowledge regarding oral health, attitudes related to oral health, oral health practices, and current guidelines and recommendations (see Supporting Information: Table S1).

### Knowledge Regarding Oral Health

Five studies^22^, ^38^, ^40^, ^42^, ^43^ discussed the oral health knowledge of women in menopause across various areas including risk factors, preventive oral hygiene measures, and the importance of oral health.

#### Risk Factors and Preventive Oral Hygiene Measures Influencing Oral Health

Overall, there was a low level of awareness among women regarding maintaining oral health during menopause.^22^, ^38^, ^40^, ^42^, ^43^ Only some women were aware of menopause being a risk factor for causing oral health problems (ranging from 1% to 53%).^22^, ^40^, ^42^, ^43^ For instance, a study conducted on 1115 women in menopause in India revealed that merely 22% were conscious of the fact that menopause may be responsible for causing gum issues.^22^ Similarly, only a few participants were aware that appropriate oral hygiene measures such as periodic teeth cleaning (19%) and regular brushing habits (32%) could prevent the loosening of teeth and periodontal problems.^22^, ^40^

### Attitudes Related to Oral Health

Among the 8 studies identified,^22^, ^32^, ^38^, ^39^, ^40^, ^41^, ^42^, ^43^ 3 reported women's attitudes toward oral health. These studies explored women's perceptions of the importance of oral health and consulting dentists.^22^, ^38^, ^42^

#### Importance of Oral Health and Consulting Dentists

There were mixed opinions among women regarding the importance of oral health. In one study by Hameed and Radhi,^38^ all women (n = 90) felt that it was their responsibility to maintain a healthy mouth. Conversely, another cross‐sectional study^22^ reported the majority (90%) of participants felt oral health could be ignored. Despite these varied attitudes, the majority of participants (69%‐100%) across 3 studies perceived regular dental visits as important, and more than half were willing to consult the dentist again.^22^, ^38^, ^42^ In one study involving 1115 women in menopause and postmenopause, approximately 80% demonstrated their willingness to attend subsequent dental visits.^22^

### Practices Relating to Oral Health

Most of the studies (n = 6) explored the practices of women in menopause in managing oral health.^22^, ^32^, ^38^, ^39^, ^40^, ^41^, ^43^ These practices included oral hygiene habits, the use of oral hygiene aids, dental visits, and barriers to accessing dental services.

#### Oral Hygiene Habits and Aids

There were limited practices in maintaining oral health among participants. Among the 5 studies that reported on oral hygiene habits and aids, it was observed that most participants brushed only once a day (30%‐84%).^22^, ^32^, ^39^, ^40^, ^41^ Within the cohort of women (n = 136) exhibiting symptoms of menopause in one study, only 16% were found to engage in brushing twice a day.^39^ The most common oral hygiene aids were toothbrushes and toothpaste (76%‐98%).^22^, ^39^, ^40^ One study observed that only 14% of individuals adhered to the practice of changing their toothbrush every 3 months.^22^ Another study reported that only 3% used more than one oral hygiene aid for maintaining oral health.^40^

#### Dental Visits

There were wide variations (range 14%‐86%) among the 6 studies that reported the frequency of dental visits.^22^, ^32^, ^38^, ^39^, ^40^, ^43^ Only 14% of postmenopausal women in Iraq (n = 61)^40^ maintained regular visits to a dentist compared with 86% among a similar demographic in the United States (n = 20).^43^ The main reasons for visits were pain (53%) and tooth concerns (71%).^32^, ^39^

#### Barriers to Accessing Dental Services

Only one cross‐sectional study from India highlighted barriers to accessing dental services for women in menopause. These included time constraints (46%), limited priority for oral health (33%), misleading advice from others about periodontal treatment (15%), and safety concerns in seeking dental care (7%).^22^

### Oral Health Knowledge of Health Care Providers

Three studies reported on oral health knowledge levels of health care providers. These studies explored their understanding of oral health in menopause with a primary focus on risk factors.^44^, ^45^, ^46^

#### Oral Health Risk Factors

There were large variations (35%‐98%) in the knowledge of various health care providers regarding menopause influencing oral health.^44^, ^45^, ^46^ The findings from a cross‐sectional study conducted across 380 medical institutions in Japan reported that nurses and primary care providers were fully aware (90%‐100%) of menopause being a risk factor for oral health.^46^ However, in another study involving gynecologists (n = 40), only 35% were knowledgeable about the link between menopause and oral health.^44^

#### Symptoms of Poor Oral Health

Only one study conducted by Rashidi Maybodi et al (2018) assessed knowledge about oral symptoms associated with menopause.^44^ A majority of the health care providers were knowledgeable in this area (90%), particularly around xerostomia (dry mouth) and thinning of mucosa as a symptom (nearly 70%). Only a few, however, were knowledgeable about reduced salivary flow (3%) and taste alterations (18%) associated with menopause.^44^

### Oral Health Attitudes of Health Care Providers

#### Treatment of Oral Symptoms and Periodic Dental Check‐ups

Health care providers were not seen as proactive in promoting oral health in one cross‐sectional study of gynecologists (n = 73) in India.^43^ More than half of the respondents (53%) did not have a positive attitude toward treating gingival symptoms associated with menopause and believed this condition would subside automatically.^45^ Most (83%) also did not feel they should recommend patients seek periodic dental check‐ups.

#### The Need for Interprofessional Collaboration

Health care providers including physicians (63%) and nurses (57%) across various medical institutions (n = 380) in Japan did agree that cooperation with dentists was necessary for managing the oral health of women in menopause.^46^ However, 76% of physicians reported that dentists never referred patients to them for medical treatment of systematic diseases, menopausal symptoms, or tooth extraction consultations.

### Oral Health Practices of Health Care Providers

#### Prevalence of Poor Oral Health and Reporting Frequency

The majority of health care providers (79%) from outpatient clinics for women in Japan highlighted that oral symptoms were reported by patients.^46^ The most commonly reported symptom was dry mouth (80%), followed by taste alterations (60%), burning sensation in the mouth (40%), and temporomandibular joint pain (20%).^46^

#### Treatment Strategies

Various treatment strategies were employed by health care providers to promote oral health.^45^, ^46^ The most common included prescribing mouthwashes, gels, antibiotics, and analgesics for the treatment of symptoms (61%); referring women to a specialist such as a dentist, otolaryngologist, or internal medicine specialist for treatment of oral symptoms (59%); documenting the referrals in health care records (48%); and informing patients about oral health changes related to menopause (44%). The less common strategies employed included prescribing medications (15%), conducting tests for Sjögren syndrome (7%), discontinuing medication (0.7%), and providing lifestyle guidance (0.7%). Only 3% of health care providers in this study provided any guidance in the treatment of oral symptoms.^46^

### Guidelines and Recommendations

Only one guideline was identified that provided recommendations for oral health care management during menopause.^47^ Although published in the United States, this guideline was developed to improve the quality of care for women worldwide. These recommendations specifically target clinical care for women in midlife and address the bodily changes that occur during this period, potential impact of hormonal fluctuations on gum, inflammation in tooth‐supporting tissues, and increased susceptibility to oral lesions. Also emphasized were recommendations for providers to advise the need for periodic dental examinations, the use of oral hygiene aids containing fluorides, maintaining good oral hygiene, and informing dental care providers about findings of various screening tests and medications use.

## DISCUSSION

The main focus of this integrative review was to assess current evidence around the oral health knowledge, attitudes, and practices among women in perimenopause and menopause, their health care providers, and the current guidelines and recommendations in this area of practice. The preliminary findings of this study indicate there is limited research undertaken in this area, particularly from the perspective of health care providers. Another interesting finding is that although high ‐income countries have better health care services and targeted strategies for health care providers to support women in menopause,^53^, ^54^ there was minimal research specifically addressing oral health needs.

Women experience various hormonal changes across their life course such as puberty, menses, reproduction, and menopause.^10^, ^55^ Although hormonal activity increases^56^ and decreases during these periods, similar oral health issues prevail.^57^ Studies pertaining to oral health have mainly focused on pregnant women and are thus the most comparable to the findings in this review.^57^, ^58^, ^59^ Overall findings from the first focus area (women's knowledge, attitudes and practices) indicated a low level of awareness during perimenopause or menopause around oral health care.^22^, ^40^, ^43^ These findings are similar to the results of a systematic review conducted on pregnant women^59^ and a cross‐sectional study conducted on menstruating women^55^ in India, which also indicated a low level of oral health knowledge among women. Additionally, there were mixed opinions regarding the importance of oral health and dental visits among women in this review, which was reflected by suboptimal oral hygiene practices and lower uptake of dental services. ^22^, ^38^, ^42^ These findings are consistent with studies from both developing and developed countries indicating women placed limited importance on oral health during pregnancy.^59^, ^60^

The lack of priority for oral health among women in perimenopause or menopause was highlighted in only one study.^22^ During this period, women undergo hormonal variations that bring about physiologic changes in their body putting them at risk for various systemic diseases.^22^ Systemic health issues may have a greater impact on quality of life, thus taking greater priority over oral health.^22^, ^61^ Time was another barrier cited.^22^ A study on the general population reported that getting time off from work or insufficient leave entitlements (especially for individuals with low income) prevented access to dental services.^62^ Similar findings were also reported in a qualitative study of pregnant women, which highlighted that caring for other children and active engagement in work prevented them from accessing dental services.^20^ Apart from the usual challenges faced by the general population and pregnant women, it is unclear why time constraints would be an issue for older women, an area that needs further exploration.

Two other contributing factors cited^22^ were safety concerns and misleading advice from others to not seek dental treatment. Although it has been well‐established that dental treatment during menopause and postmenopause is completely safe,^22^ similar concerns have been highlighted among pregnant women questioning the safety and effects of dental treatment on the fetus.^21^ Equally concerning were reports of receiving misleading advice from others regarding the safety of dental care or procedures. Conflicting advice has been documented among prenatal care providers during interactions with pregnant women. Research exploring the perceptions and practices of prenatal care providers regarding oral health care found that some primary care providers believed dental procedures were unsafe during pregnancy, particularly the use of anesthesia and radiographs.^63^ Further research is needed to better understand if women in perimenopause and menopause are being provided the correct oral health advice from their health care providers.

It is evident from this integrative review that only some health care providers are informing women about oral health changes associated with menopause, and even fewer are providing guidance on treating oral symptoms. Likewise, there has been a limited focus on preventive strategies such as screening and oral health promotion. Studies have also suggested that women require comprehensive care during this period, but often, health care providers are unable to spend more time due to barriers such as underfunding and staff shortages.^53^ Furthermore, reviews around women's health issues worldwide found that primary care providers and midwives were poorly informed about the impact of poor maternal oral health and rarely initiated this topic during prenatal care.^64^ Only 22% of prenatal care providers in a study conducted in Turkey discussed the importance of oral health with pregnant women.^63^

The are numerous factors that could be contributing to the limited focus of oral health during perimenopause and menopause by health professionals. For example, only one clinical practice guideline developed by the North American Menopause Society (NAMS) was identified.^47^ In addition to providing valuable information on the impact of hormonal changes on oral health, the NAMS guideline recommends health professionals provide oral health education, screening, and referrals through collaborations with dental practitioners.^46^ In a survey of 393 antenatal care providers in Australia involving primary care providers, midwives, and obstetricians, 81% cited lack of practice guidelines as the main barrier to promoting oral health during pregnancy.^29^ More work is clearly needed to develop global evidence based oral health guidelines for health professionals supporting women in perimenopause or menopause.

It is important to note that there may be other contributing factors limiting health professionals from promoting oral health among women in perimenopause and menopause. Previous studies involving primary care providers, nurses, midwives, and allied health professionals have highlighted a lack of oral health knowledge and confidence, time constraints, and limited oral health screening tools.^29^, ^65^ Additionally, issues around the cost and accessibility of dental care are often cited as barriers to oral health care by various population groups at risk for poor oral health such as those with diabetes, mental illness, and cardiovascular disease.^21^, ^66^ Larger studies and more in‐depth high‐quality research is needed to explore these aspects further.

### Implications for Practice

A main finding from this review is the need for governments and professional organizations to develop appropriate clinical practice guidelines for health care providers that promote oral health among women in perimenopause or menopause. Additional oral health training could also be provided to health care providers via professional development training programs and undergraduate modules to improve awareness of the importance of oral health in this population group. Lastly, interprofessional collaboration between health care providers and dental practitioners should be encouraged to improve the oral health literacy of women in perimenopause and menopause with appropriate dental referral pathways.

A number of health professionals who participated in studies included in this review felt that interprofessional collaboration between health care and dental providers was important for promoting oral health care of women.^46^ Numerous studies have shown that adopting such an approach can deliver improved patient outcomes and sustainable integrated models of care.^67^, ^68^, ^69^ One such example is the Midwifery Initiated Oral Health (MIOH) program developed in Australia to provide knowledge and skills to midwives to promote oral health in pregnant women.^70^ Through this program, midwives are trained to work collaboratively with dental practitioners to provide oral health education, screening, and referrals with the help of a continuing professional development training program (endorsed by the Australian College of Midwives) and a simple validated screening tool.^24^ The MIOH program has been shown to be effective in improving the oral health knowledge and confidence of midwives as well as the oral health status and quality of life of pregnant women, and is cost‐effective for health services. The program is recognized by the World Health Organization and has been implemented across Australia and internationally.^70^, ^71^

### Strengths and Limitations

To our knowledge, this review is the first to evaluate current evidence on oral health knowledge, attitudes, practices, and guidelines. These results have provided valuable insight into this underresearched area as well as a roadmap for future research.

It is also important to acknowledge the study limitations. Because only a limited number of studies were identified for this review, and the majority were of poor quality, the study findings and conclusion must be interpreted with caution. Furthermore, most of the studies were from low‐income countries, and thus the findings may not be applicable in all geographical areas. Moreover, standard practice patterns and guidelines in this area may differ in higher resource countries.

## CONCLUSIONS

This integrative review has provided a valuable insight in the oral health care of women in perimenopause or menopause. Overall, the findings suggest that there is a lack of oral health awareness and poor oral hygiene practices among women during this life transition. Furthermore, oral health needs are not adequately addressed by health care providers due to various barriers. Practice guidelines are needed to address oral health needs and improve oral health services. These results point out an urgent need for further high‐quality research to confirm findings from both the women's and health care providers’ perspectives and inform supportive strategies across policy and practice in this area.

## CONFLICT OF INTEREST

The authors have no conflicts of interest to disclose.

## Supporting information


**Table S1**. Themes and Subthemes
