# Supplementary material for: Oral Health Care Among Women in Perimenopause or Menopause: An Integrative Review
Source: J Midwifery Womens Health. 2024 Jul 24;70(1):17–31. doi: 10.1111/jmwh.13668 (PMC11803492; doi:10.1111/jmwh.13668)
Supplement: Supplementary file 1 — Table S1. Themes and Subthemes [file JMWH-70-17-s001.docx]

**Table S1. Themes and Subthemes**

| **Themes and Subthemes** | **Data Contributing to Themes** |
| --- | --- |
| **Oral health knowledge** |  |
| **Women** |  |
| Risk factors | Singh & Jamwal (2020)^[22](#_ENREF_22)^; Gatchalian et al (2022)^[42](#_ENREF_47)^; Palomo et al (2013)^43^; |
| Preventive oral hygiene measures influencing oral health | Singh & Jamwal (2020)[^22^](#_ENREF_22); Hameed & Radhi (2023)^38^; Qasim et al (2017)^40^ |
| **Health care providers** |  |
| Symptoms of poor oral health | Rashidi Maybodi et al,(2018)^44^; |
| Oral health risk factors | Rashidi Maybodi et al,(2018)^44^ ; Patil et al, (2012)^45^; Matsuki et al, (2013)^46^ |
| **Oral health attitudes** |  |
| **Women** |  |
| Consulting Dentist | Singh & Jamwal (2020)[^22^](#_ENREF_22); Hameed & Radhi (2023)^38^; Gatchalian et al (2022)^42^ |
| Importance of oral health | Singh & Jamwal (2020)[^22^](#_ENREF_22) |
| Barriers to accessing dental services | Singh & Jamwal (2020)[^22^](#_ENREF_22) |
| **Health care providers** |  |
| Treatment of oral symptoms | Patil et al, (2012)^45^ |
| Periodic dental check-ups | Patil et al, (2012)^45^ |
| The need for interprofessional collaboration | Matsuki et al, (2013)^46^ |
| **Oral health practices** |  |
| **Women** |  |
| Oral hygiene habits and aids | Singh & Jamwal (2020)[^22^](#_ENREF_22); Qasim et al (2017)^40^; Basu et al (2021)^39^; Yakar et al (2021)[^32^](#_ENREF_32); Deepa & Jain (2016)^41^ |
| Dental visits | Singh & Jamwal (2020)^[22](#_ENREF_22)^; Qasim et al (2017)^40^; Basu et al (2021)^39^; Yakar et al (2021)^[32](#_ENREF_32)^; Hameed & Radhi (2023)^38^; Palomo et al (2013)^43^ |
| Dental visits -reasons | Basu et al (2021)^39^; Hameed & Radhi (2023)^38^ |
| **Health care providers** |  |
| Treatment strategies | Patil et al (2012)^45^; Matsuki et al (2013)^46^ |
| Frequency of reporting oral symptoms | Matsuki et al (2013)^46^ |
| Prevalence of poor oral health | Matsuki et al (2013)^46^ |
| **Guidelines** |  |
| Recommendations for clinicians | Shifren et al (2014)^47^ |
